# Protein encapsulation within the internal cavity of a bacterioferritin†

**DOI:** 10.1039/d2nr01780f

**Published:** 2022-08-15

**Authors:** Justin M. Bradley, Elizabeth Gray, Jake Richardson, Geoffrey R. Moore, Nick E. Le Brun

**Affiliations:** Centre for Molecular and Structural Biochemistry, School of Chemistry, University of East Anglia, Norwich Research Park Norwich NR4 7TJ UK Justin.bradley@uea.ac.uk n.le-brun@uea.ac.uk; Bioimaging Facility, John Innes Centre, Norwich Research Park Norwich NR4 7TJ UK

## Abstract

The thermal and chemical stability of 24mer ferritins has led to attempts to exploit their naturally occurring nanoscale (8 nm) internal cavities for biotechnological applications. An area of increasing interest is the encapsulation of molecules either for medical or biocatalysis applications. Encapsulation requires ferritin dissociation, typically induced using high temperature or acidic conditions (pH ≥ 2), which generally precludes the inclusion of fragile cargo such as proteins or peptide fragments. Here we demonstrate that minimizing salt concentration combined with adjusting the pH to ≤8.5 (*i.e.* low proton/metal ion concentration) reversibly shifts the naturally occurring equilibrium between dimeric and 24meric assemblies of *Escherichia coli* bacterioferritin (Bfr) in favour of the disassembled form. Interconversion between the different oligomeric forms of Bfr is sufficiently slow under these conditions to allow the use of size exclusion chromatography to obtain wild type protein in the purely dimeric and 24meric forms. This control over association state was exploited to bind heme at natural sites that are not accessible in the assembled protein. The potential for biotechnological applications was demonstrated by the encapsulation of a small, acidic [3Fe-4S] cluster-containing ferredoxin within the Bfr internal cavity. The capture of ∼4–6 negatively charged ferredoxin molecules per cage indicates that charge complementarity with the inner protein surface is not an essential determinant of successful encapsulation.

## Abstract 2

The controlled, reversible dissociation of bacterioferritin allows the trapping of guest molecules such as proteins within the internal cavity.

## Graphical abstract

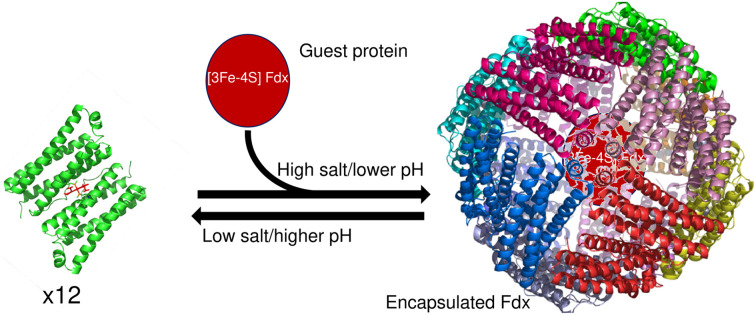

## Introduction

Proteins that self-assemble to yield cage-like topologies have attracted considerable interest due to their potential applications in biotechnology. The defined, homogenous environment of their interiors may offer superior control of nanoscale fabrication processes in engineered bioreactors.^1–3^ Furthermore, the encapsulation of pharmacologically active molecules within protein cages raises the possibility of their exploitation as vehicles for drug delivery.^4,5^ Protein-based cages offer several advantages over their inorganic or organic counterparts produced by chemical synthesis; chief among these are greater efficiency of cellular uptake and the ease of functionalization offered by genetic control.^6–8^ Natural cage-forming systems with reported potential applications in biotechnology include virus capsids,^1,9^ eukaryotic vaults,^10^ bacterial microcompartments,^11^ lumazine synthase capsids,^12^ encapsulins^3,13,14^ and ferritins.^6,7,15–17^

Ferritins are ubiquitous proteins involved in iron homeostasis and alleviation of oxidative stress.^18^ During normal function, oxidation of iron is coupled to the reduction of O_2_ or peroxide, leading to the deposition of a ferric-oxyhydroxide mineral within an internal cavity.^19^ This approximately spherical cavity is 80 Å in diameter and delimited by the self-assembly of 24 protein monomers. These 24-meric assemblies display remarkable thermal and chemical stability that, together with the presence of both hydrophilic and hydrophobic channels penetrating the protein coat,^20^ make ferritins excellent candidates for biotechnology applications. Their ability to oxidise iron has been exploited in conjunction with their stability over relatively large ranges of temperature, pH and chemical conditions to lay down non-native cores with applications in bio-imaging.^14^ Other biotechnological applications, including encapsulation of small molecule drugs for medical intervention or proteins for biocatalysis, require a strategy for the controlled dissociation and re-association of the multimeric protein assembly, or to otherwise render the protein coat permeable to the molecule(s) of interest.^15–17,21–23^

**Fig. 1:**
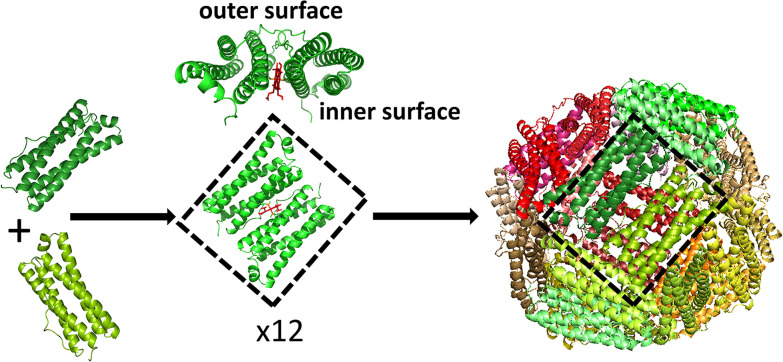
Assembly of Bfr. The first step in the assembly process is thought to be the association of two 4 α-helical bundles to produce the subunit dimer. The long axes of the two protomers are arranged in an anti-parallel orientation with a binding site for heme at their interface. Axial ligation of the heme iron is provided by the sidechain of Met52 from each protomer, leading to positioning of the heme on what would constitute the inner surface of the fully assembled cage.

Ferritin assembly is thought to proceed *via* the association of dimeric units into higher order oligomers,^24–27^ eventually leading to formation of the rhombic dodecahedral 24mer in which subunit dimers make up each of the 12 faces (Fig. 1). The inherent stability of ferritin dimers has allowed high temperature and/or extremes of pH to be used to disassemble protein cages without denaturing the fold of the individual monomers. Self-assembly on return to milder conditions then allows encapsulation of exogenous molecules within the re-formed ferritin shell, but recovery of reassembled protein can be as low as 10%.^7,28^ Furthermore, the use of harsh conditions to induce ferritin dissociation restricts the range of guest molecules for which encapsulation is possible. An alternative approach is to use targeted mutagenesis to produce variant proteins in which the association state can be controlled by, for example, the addition of copper ions.^29^

Whist the use of human ferritins as image contrast agents or drug delivery vectors offers the obvious advantage of reduced immunogenicity,^30^ the use of ferritins from other sources has also been pursued. The ferritins (Ftns) from the archaeon *Archaeoglobus fulgidus* and the anaerobic hyperthermophile bacterium *Thermotoga maritima* are unusual in that they do not exhibit the high stability of the assembled form that is typical of most 24mer ferritins: they form an equilibrium mixture of 24mer and (probably) dimeric forms, and the position of the equilibrium can be shifted to either extreme by changes in ionic strength/metal ion concentration.^15,17,31,32^

While *T. maritima* Ftn adopts a structure typical of animal and bacterial 24mer ferritins, with octahedral symmetry, that of *A. fulgidus* adopts a quite different structure, with tetrahedral symmetry that results in large 45 Å diameter pores through the protein coat, which may limit its suitability as a delivery vector or nanocompartment. Nevertheless, encapsulation of positively charged proteins (such as lysozyme and a supercharged, +36 variant of GFP and fusions of it with various enzymes) within these Ftns could be achieved by simply mixing the ferritin with the basic protein and incubating overnight. Electrostatic charge complementarity appears to be crucial for successful encapsulation, because less charged (+10 GFP) or negatively charged GFP were not encapsulated.^15,17,32^ Furthermore, interchange of the oligomeric forms of both proteins appears to be rapid such that complementary charge was also required for retention of the cargo molecules, as evidenced by a decrease in the efficiency of encapsulation in buffer with high ionic strength. As a result these systems may be prone to leakage of encapsulated cargo at moderate to high salt concentrations (0.3 M and above).

Bacterioferritins (Bfrs) are a distinct class of ferritin cages, found only in bacteria, that contain a heme prosthetic group at the interface of each subunit dimer.^33^ It has long been known that some Bfr proteins naturally exist in equilibrium between the subunit dimer and the fully assembled cage.^34,35^ In the case of the protein from *Escherichia coli*, this is thought to arise, at least in part, because of the presence of water filled pockets at the interface between dimers in the assembled cage.^36^ This reduces the size of the buried hydrophobic area at the 2-fold and 4-fold axes formed on association of dimers relative to that in other ferritins, thereby destabilising the 24meric cage. The relatively small energy difference between the different association states of *E. coli* Bfr suggests that assembly and disassembly of the cage may be induced through more subtle changes in conditions than those required for the animal ferritins, offering the possibility of encapsulation of molecules under benign conditions and with a greater degree of protein recovery, as reported for the two Ftns.

Here we report a systematic investigation into the effect of pH and metal ion concentration on the equilibrium between fully assembled Bfr cages and the subunit dimer and demonstrate that this can be altered in favour of the latter by employing buffer systems with moderately high pH and low ionic strength. Interchange between the oligiomeric forms is slow, allowing the isolation of purely dimeric wild type Bfr by size exclusion chromatography. That this strategy allows exogenous molecules to access the inner surface of the protein cage was demonstrated by *in vitro* loading of heme, the binding site for which is inaccessible from the exterior of the assembled protein. The discovery of mild conditions promoting Bfr dissociation was subsequently exploited to trap a small (approximately 8 kDa), acidic ferredoxin within the protein cage. The net negative charge of the encapsulated ferredoxin indicates that charge complementarity with the inner surface of the ferritin is not required for encapsulation within *E. coli* Bfr. Spectroscopic measurements indicated that the ferredoxin iron-sulfur cluster remained intact following the encapsulation procedure. This work extends the range of potential cargoes that can be encapsulated with cage forming proteins.

## Experimental

### Protein expression and purification

Wild type *E. coli* Bfr was produced in *E. coli* strain BL21 DE3 (Promega) as described elsewhere.^37^ Briefly, cultures were grown at 37 °C, 200 rpm shaking to an optical density at 600 nm of 0.6–0.8 absorbance units. Protein expression was then induced by addition of isopropyl β-d-1 thiogalactopyranoside (IPTG) to a final concentration of 10 μM. Cultures were incubated for a further three hours prior to harvesting by centrifugation. Pelleted cells were re-suspended in 20 mM HEPES, 100 mM KCl, 0.1 mM EDTA, pH 7.8 (buffer A), prior to disruption by sonication. Cell debris was removed by centrifugation and thermally unstable proteins precipitated by heating to 65 °C for 15 min. Following a further round of centrifugation, Bfr was precipitated from solution by addition of ammonium sulfate to a concentration of 0.55 g mL^−1^. The pelleted protein was re-dissolved in buffer A and dialysed against identical buffer for a minimum of 12 h. Contaminating proteins were removed by size exclusion chromatography (HiPrep 26/60 Sephacryl S-300HR, Cytiva) and contaminating DNA by anion exchange chromatography (HiTrap Q FF, Cytiva). For the latter, protein solutions were loaded in buffer A and eluted by stepping to 50% buffer B (20 mM HEPES pH 7.8 containing 100 mM KCl, 1.0 M NaCl, 0.1 mM EDTA).

Protein as isolated contained small quantities of iron that were removed as previously described,^38^ and protein subsequently exchanged into 100 mM MES pH 6.5 by centrifugation over a 10 kDa molecular weight cut off cellulose membrane (Millipore). Sample purity was assessed using SDS-PAGE and protein judged to be free of DNA contamination once the ratio of absorbance at 280 nm over 260 nm reached 2.0. Protomer concentration was determined by absorbance^39^ using *ε*_280 nm_ = 3.33 × 10^4^ M^−1^ cm^−1^.

Plasmid DNA encoding the gene Mmar_3973, with C-terminal His tag incorporated in a pETDuet vector, was provided by Dr Stephen Bell (University of Adelaide). Cultures were grown at 37 °C, 200 rpm shaking until the optical density at 600 nm was within the range 0.6–0.8, prior to cold shocking on ice for 18 min. Protein expression was initiated by addition of IPTG to a final concentration of 50 μM and cultures supplemented with ammonium ferric citrate and l-cysteine (20 μM), before growing on for a further 20 h at 25 °C, 90 rpm shaking. Cells were harvested by centrifugation and re-suspended in anaerobic buffer A (20 mM HEPES, 100 mM NaCl, 20 mM imidazole, pH 7.4), ruptured by sonication and debris removed by centrifugation (40 000*g* for 45 min) under nitrogen in gas tight tubes. All subsequent steps were carried out under a nitrogen atmosphere in an anaerobic chamber (Belle Technology, [O_2_] < 10 ppm). Supernatant was loaded onto a Ni^2+^ charged IMAC column (Cytiva) equilibrated with buffer A and eluted using a gradient from 0 to 100% of buffer B (as buffer A but containing 500 mM imidazole). Ferredoxin-containing fractions (as identified by dark brown colour) were exchanged into 100 mM MES pH 6.0 containing 3 M NaCl as cryoprotectant using a PD-10 column and stored at −5 °C under an anaerobic atmosphere prior to use. Mmar_3973 ferredoxin was quantified (in terms of cluster) by absorbance^40^ using *ε*_410 nm_ = 9000 M^−1^ cm^−1^.

### Determination of Bfr association state

The observation that the equilibrium between dimeric and 24meric Bfr is dependent on the concentration of cationic species precluded the use of gel electrophoresis under non denaturing conditions to assess the association state of the protein. Rather, the oligiomeric state was determined by analytical gel filtration chromatography. 300 μL aliquots of protein (500 μM monomer concentration) were exchanged into the desired buffer by centrifugation over a 3 kDa membrane (Millipore) and allowed to equilibrate for 16 h at 4 °C before eluting from a Superdex 75 10/300 GL column (Cytiva) that had been pre-equilibrated with identical buffer. Based on a column calibration performed using proteins of known molecular weight (Sigma kit for molecular weights 12–200 kDa), it was predicted that the elution volume of the fully assembled protein would be 8 mL and that of the subunit dimer 11.5 mL.

### Heme loading of Bfr

The capacity of Bfr to bind heme under selected conditions was probed by absorbance spectroscopy (spectra recorded using a Jasco V550 spectrophotometer) upon titration with freshly prepared solutions of hemin chloride. Hemin chloride (Sigma) was dissolved in a minimum volume of 100 mM NaOH before diluting in 100 mM Tris pH 9.0 to a final concentration of 250 μM (concentration estimated using *ε*_385 nm_ = 5.9 × 10^4^ m^−1^ cm^−1^). 5 μL aliquots of this solution were added to 2 mL of 36 μM Bfr (protomer concentration) in the desired buffer and the degree of heme binding assessed by the increase in absorbance at 417 nm due to Bfr-associated low-spin ferric heme.

### Encapsulation of ferredoxin Mmar_3973

Bfr was exchanged into 20 mM Tris pH 9.0 (buffer 1) and stored overnight at 4 °C before being subjected to size exclusion chromatography using a column equilibrated with identical buffer. The fraction eluting at 11.5 mL was diluted to a concentration of 1 mg mL^−1^ and 0.5 mL transferred to an anaerobic chamber prior to mixing with an equal volume of Mmar_3973 ferredoxin (10 mg mL^−1^) in 100 mm MES, 3 M NaCl pH 6.0 (buffer 2), such that the resulting solution had pH ∼6.5 and contained 1.5 M NaCl. Samples were incubated at 25–30 °C for 3–4 hours to allow re-assembly of the Bfr cage. Ferredoxin not encapsulated within reformed Bfr cages was removed by manual loading of the sample onto a Ni^2+^ charged IMAC column. Bfr together with any encapsulated Mmar_3973 eluted when washing the column with 20 mM HEPES, 100 mM NaCl, 20 mM imidazole, pH 7.4. Ferredoxin not encapsulated within the Bfr cage bound to the IMAC column *via* the C-terminal His tag and was subsequently eluted by stepping to 100% of buffer containing 500 mM imidazole. Samples were exchanged into 100 mM MES containing 1.5 M NaCl under anaerobic conditions and the protein content of each fraction confirmed using ESI-LC-MS. Absorbance spectra of encapsulated ferredoxin were recorded on a Jasco V550 spectrophotometer to confirm that the [3Fe-4S] cluster remained intact during this process.

### Liquid chromatography mass spectrometry (LC-MS)

ESI-LC-MS was performed using an UltiMate 3000 HPLC system (Dionex). Briefly, 25 μL of each sample was diluted with 475 μL LC-MS buffer (2% (v/v) acetonitrile, 0.1% (v/v) formic acid), and 15 μL of each sample was injected onto a ProSwift reversed phase RP-1S column. A gradient elution was performed at a flow rate of 0.2 mL min^−1^ using a linear gradient of solvent A (0.1% (v/v) formic acid) and solvent B (acetonitrile with 0.1% (v/v) formic acid), from 2% solvent B to 100%, controlled through the Hystar software. The eluent was directly infused into a Bruker micrOTOF-QIII mass spectrometer (Bruker Daltonics), and mass spectra were acquired between 50–3000 *m*/*z* with the following parameters: dry gas flow 8 L min^−1^, nebuliser gas pressure 0.8 bar, dry gas temperature 240 °C, capillary voltage 4500 V, offset 500 V, collision RF 650 Vpp. Mass spectra from manually chosen elution volumes were averaged and deconvoluted over the mass range 5–26 kDa.

### Transmission electron microscopy

3.5 μL of sample was placed on a carbon, 400 mesh copper grid (Agar Scientific Ltd, Stansted, UK) that had been glow discharged for 20 seconds at 10 mA in an Ace 200 (Leica Microsystems (UK) Ltd, Milton Keynes, UK). Excess sample was wicked away using Whatman No. 1 filter paper after 60 seconds, and grids washed twice by successively floating on droplets of water. Excess water was wicked away and grids negatively-stained using 2% (w/v) uranyl acetate in water for 30 seconds, excess wicked then allowed to air dry. Grids were imaged using a Talos F200C transmission electron microscope (Thermo Fisher Scientific, Eindhoven, The Netherlands) operated at 200 kV, and equipped with a 4k OneView CMOS detector (Gatan UK, Abingdon, Oxfordshire, UK).

## Results

### Bfr association state is influenced by both pH and metal ion concentration

**Fig. 2:**
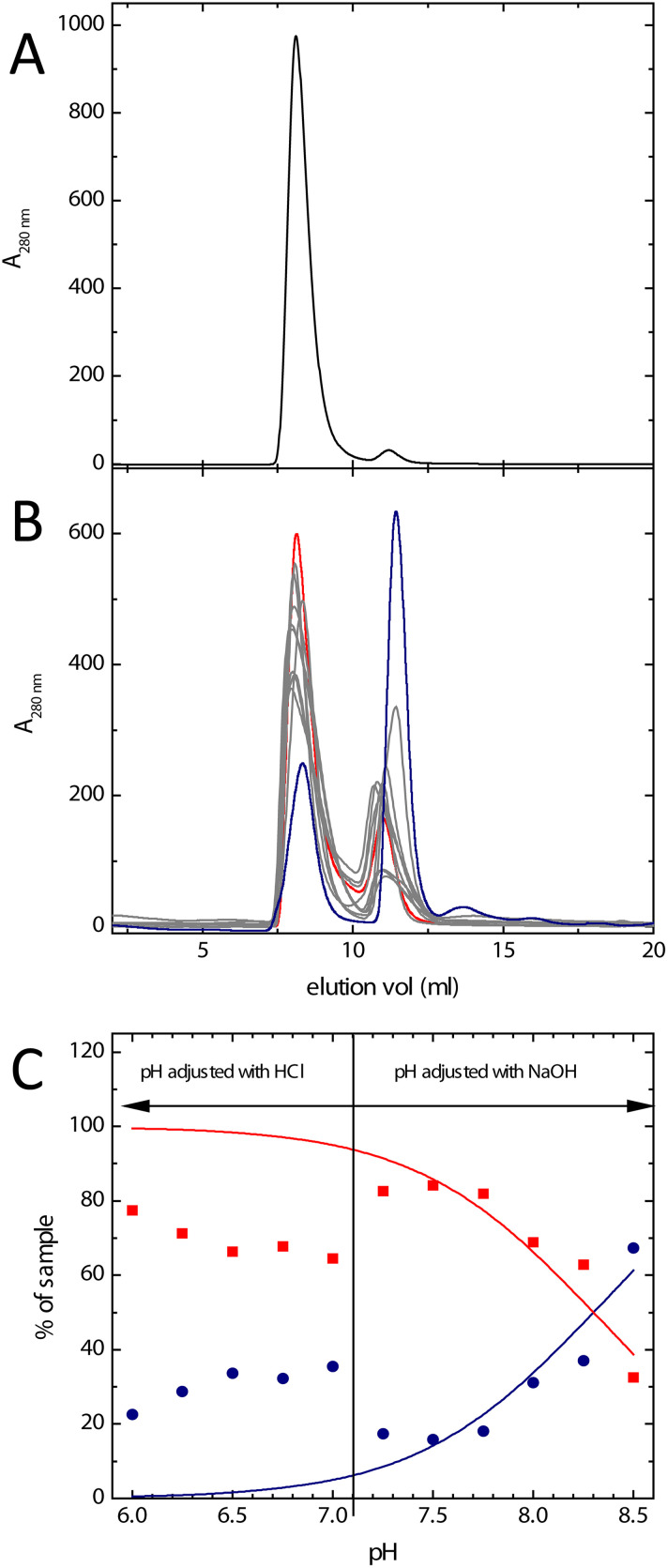
The effect of pH on Bfr association state. (A) The elution profile of Bfr in 100 mM MES pH 6.5 from a Superdex 75 10/300 GL analytical gel filtration column equilibrated with identical buffer. The major peak corresponds to the predicted elution volume of the fully assembled protein cage with the smaller feature eluting at the larger volume predicted for the subunit dimer. (B) As (A) but with Bfr and column equilibrated in a mixed buffer system across the pH range 6.0 to 8.5. The heavy red trace represents the sample equilibrated at the lowest pH and the heavy blue trace that at the highest. Samples equilibrated at intermediate pH are represented by light grey traces. (C) The proportion of Bfr present as fully assembled cages (red squares) or subunit dimer (blue circles) as a function of pH estimated by integrating the area under the elution peaks shown in (B). The solid lines represent the predicted proportion of fully assembled protein (red) or subunit dimer (blue) for dissociation caused by a single de-protonation event with a p*K*_a_ of 8.50.

The earlier observation of discrete bands corresponding to dimeric and fully assembled Bfr in non-denaturing polyacrylamide gel electrophoresis^34,35^ demonstrated the inter-conversion of these oligomeric states to be slow or non-existent under the conditions of the experiment. Either of these possibilities is compatible with separation of the different Bfr association states by size exclusion chromatography. The chromatogram obtained in the buffer system commonly employed for kinetic and mechanistic studies of Bfr (100 mm MES, pH 6.5) demonstrated that, as expected, the protein existed predominantly (approximately 95% of the sample) in the 24meric form (Fig. 2A). Fractions containing either dimeric or fully assembled protein were pooled separately and stored at 4 °C. 300 μL aliquots of these pooled fractions were used to monitor any changes in association state over several days. The data showed that, under these conditions, the distribution of oligomeric forms shifted back towards that at equilibrium, but was very slow (Fig. S1†). This demonstrated that, firstly, assembly of the 24mer from the dimer is a spontaneous process requiring no additional co-factors, and secondly, that size exclusion chromatography represents a viable method of separating different association states, and of obtaining Bfr almost exclusively in its dimeric form.

High pH has been shown previously to favour the dissociation of 24mer Bfr into subunit dimers.^35^ A mixed buffer system comprised of MES, HEPES and Tris (all at 20 mM) was employed to investigate the effect of pH over the range 6.0–8.5. The resulting chromatograms are shown in Fig. 2, together with the estimated proportion of dimeric and fully assembled protein as a function of pH. The data showed the expected general trend of an increase in the extent of dissociation into subunit dimer with increasing pH, with over 50% of the total protein content of the sample being in the dimeric form at pH 8.5. The degree of dissociation in the mixed buffer system at pH 6.5 was greater than that observed in 100 mM MES (Fig. 2) and there was a marked discontinuity between pH 7.0 and 7.25. This corresponds to the ambient pH of the mixed buffer system, and therefore the point at which NaOH needed to be added in order to attain the desired pH for measurement. This suggests that the presence of Na^+^ ions favours assembly of the protein cage.

**Fig. 3:**
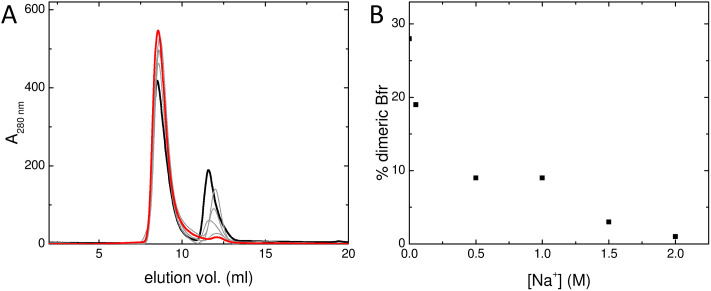
Effect of Na^+^ on Bfr association state. (A) the elution profile of Bfr in mixed buffer at pH 8.0 at various [Na^+^] between 0 and 2 M from a Superdex 75 10/300 GL analytical gel filtration column equilibrated with identical buffer. The heavy black trace depicts the elution profile in the absence of added NaCl and the heavy red trace that in the presence of 2.0 M NaCl. Elution profiles at intermediate [NaCl] are shown as grey lines. (B) The proportion of each of the samples from (A) present as subunit dimer as estimated from the integrated peak areas in the chromatograms.

Therefore, the effect of Na^+^ on the association state of Bfr was investigated. Samples of Bfr were exchanged into the mixed buffer system at pH 8.0 at a range of NaCl concentrations from 0.05–2.0 M, and the association state investigated using size exclusion chromatography, as above. The resulting chromatograms (Fig. 3) demonstrated that, as predicted, increasing concentration of Na^+^ favoured full assembly of the cage over subunit dimers.

### *In vitro* heme loading of Bfr

Bacterioferritins are distinct from all other ferritins in that they bind a heme group located at the interface between the two protomers of each subunit dimer (Fig. 1). This results in a characteristic absorbance centred around 417 nm due to the Soret peak of the oxidized heme. In addition, the highly unusual Met/Met axial ligation of the heme iron gives a ligand to metal charge transfer band at around 742 nm.^41^ The heme binding site is located on the inner surface of assembled cages, rendering it inaccessible to exogenously added heme, since this prosthetic group is too large to traverse the channels penetrating the protein. It was previously shown that treatment of the protein at high temperature (80 °C) facilitates uptake of heme.^42^ In contrast, the heme binding sites of an engineered subunit dimer of *E. coli* Bfr, which is not capable of association to form the 24meric protein cage, are readily accessible and bind heme with high affinity.^43^ Furthermore, due to inefficient heme synthesis in aerobically respiring *E. coli* cells, overexpressed Bfr protein is deficient in heme as isolated, and typically contains on average only 1.0–1.5 of the possible 12 hemes per protein cage.^44^ Therefore, the capacity of Bfr to bind added heme serves as a convenient model system with which to investigate the possibility of employing controlled dissociation and association of the cage for encapsulation of biological molecules.

**Fig. 4:**
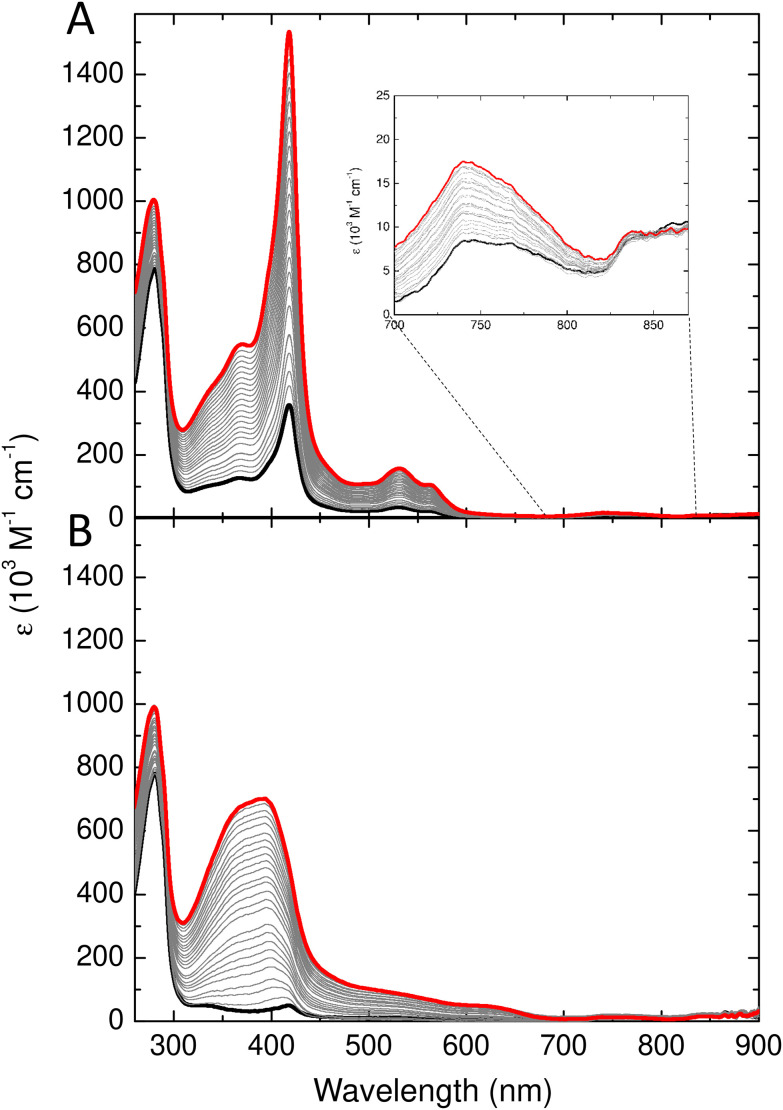
Titration of Bfr with hemin. UV-visible absorbance spectra following titration of hemin into (A) the dimeric fraction obtained from analytical gel filtration of Bfr equilibrated with 20 mM Tris pH 8.5, and (B) Bfr in 100 mM MES pH 6.5. The heavy black traces represent the spectra of samples prior to addition of hemin and heavy red traces the end points of the titrations. Intermediate spectra are shown in grey. The inset to (A) shows the region around 740 nm on an expanded scale.

Wild type Bfr was exchanged into 20 mM Tris at pH 9.0 and subjected to size exclusion chromatography as above but with the column equilibrated with Tris. The fraction containing dimeric Bfr (expected to elute at 11.5 mL) was then diluted to a concentration of 36 μM Bfr protomers (18 μM in heme binding sites) and a 2 mL aliquot transferred to a 1 cm pathlength quartz cuvette for spectroscopic determination of heme binding. Fig. 4A shows the change in absorbance following successive additions of hemin chloride. The systematic increase in absorbance at 417 nm showed that the ferric iron in the added hemin adopts a low spin ferric electronic configuration, as a high-spin configuration results in a Soret band below 400 nm (compare Fig. 4A and B). The low spin configuration is a result of heme binding at the dimer interface, and the increased 742 nm absorbance is diagnostic of at least a sub-population of the added heme adopting the native Met/Met axial ligation. In contrast, the iron of hemin chloride in solution adopts a high spin electronic configuration, resulting in major absorbance features at 384 and 613 nm. Titration of hemin chloride into a solution of as purified Bfr in MES pH 6.5, in which approximately 95% of the protein exists as 24meric cages, resulted in increased absorbance at 392 and 624 nm, with no significant increase in intensity of the features at 417 and 742 nm (Fig. 4B). These observations confirm that fully assembled Bfr cages are incapable of binding hemin at the native inter-subunit sites and, therefore, that in Tris at pH 9.0 the majority of the protein remains in the dimeric form over the several hours required for the heme binding experiment.

### Reversible disassembly/reassembly of Bfr cages

Lowering the concentration of salt in buffered solutions at a pH above neutral promotes dissociation of the protein cage into constituent dimeric units. However, an effective encapsulation strategy also requires a means of returning the protein to a fully assembled state. To this end, heme-loaded dimeric Bfr was exchanged into MES pH 6.5 containing 1.5 M NaCl, in an attempt to reverse the dissociation induced by low salt concentration and elevated pH. The protein was then exchanged in to 100 mM MES pH 6.5 with no additional NaCl added to enable comparison with samples that had not been subjected to the protocol for dissociation of the cage. Fig. S2† shows the elution profile from an analytical size exclusion column of protein treated in this way, demonstrating that virtually all of the sample was returned to the 24meric state. Re-cycling of Bfr eluting as 24mer and allowing the equilibrium population to be re-established prior to further rounds of size exclusion chromatography resulted in approximately 70% yield of protein following the disassembly/reassembly process.

### Encapsulation of a protein within the Bfr cage

Having established a protocol for the controlled dissociation and re-assembly of the Bfr protein cage, we sought to demonstrate encapsulation of a molecule that would not be possible *via* the previously reported acid dissociation route. As proof of principle, we chose the electron transfer ferredoxin encoded by gene Mmar_3973 of *Mycobacterium marinum*. The protein can be overexpressed in high yield from *E. coli* using a plasmid construct that encodes the protein with a C-terminal His tag. The protein is relatively small (7646 Da for the apo-protein), and would therefore be easily accommodated within the internal cavity of Bfr. No structures of the protein are available but the similarly sized ferredoxin from *Rhodopseudomonas palustris* (PDB 4ID8) has approximate dimensions of 30 × 26 × 20 Å and would therefore occupy 6% of the volume within the 80 Å diameter Bfr internal cavity.

Encapsulation of the ferredoxin within Bfr was attempted using an adaptation of the protocol used for capture of the small molecule doxorubicin within human H-chain ferritin.^45^ In brief, this involved disassembly of Bfr into subunit dimers (using buffer 1, see Methods) and addition of an excess of the ferredoxin (in buffer 2, see Methods) under conditions that promote reassembly of Bfr. Solutions were incubated for a period of several hours prior to separation of the proteins using either Ni^2+^ affinity or size exclusion chromatography. Previous reports of protein encapsulation within ferritin cages have typically employed negative stain transmission electron microscopy (TEM) to demonstrate exclusion of uranyl acetate from the interior of the ferritin cage by the captured cargo.^15,17^ Whilst TEM demonstrated the expected reassembly of the Bfr cage (Fig. S2†), the small size of the ferredoxin utilised here rendered such an approach impractical. Rather, evidence of encapsulation was provided by analysis by LC-MS of fractions collected during chromatographic separations of Bfr/ferredoxin mixtures. Firstly, the wash and elution fractions eluting from a Ni^2+^ affinity column were analysed to determine their protein content. Control samples prepared by mixing dimeric Bfr with buffer 2, and mixing Mmar_3973 with buffer 1, confirmed negligible interaction of Bfr with the Ni^2+^ affinity column and negligible flow through of non-encapsulated ferredoxin, respectively (Fig. S3†).

**Fig. 5:**
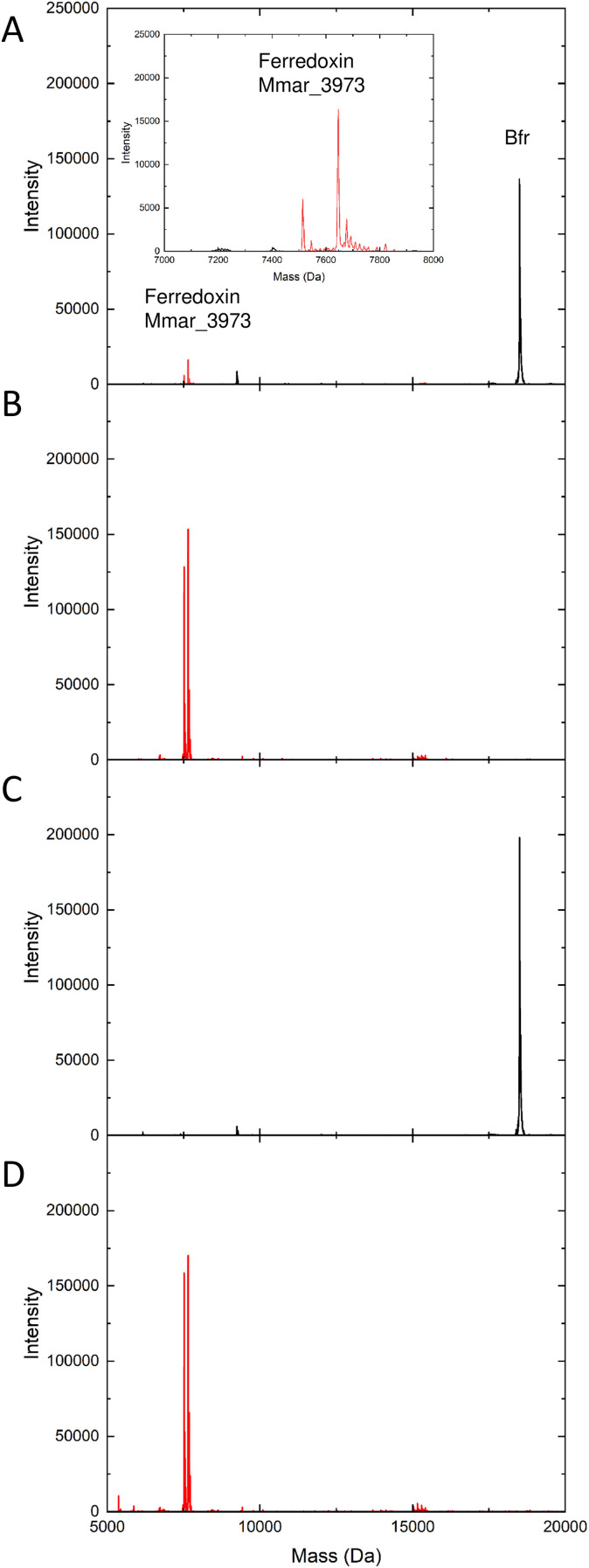
Investigation of ferredoxin encapsulation with Bfr using LC-MS. Identification of the protein masses present in fractions eluting from a Ni^2+^ affinity column following the mixing of equal volumes of Bfr and the ferredoxin Mmar_3973. Mass spectra shown are from samples collected during washing of the column with binding buffer (A) and elution of bound material after stepping the buffer imidazole concentration to 500 mM (B) following the mixing of dimeric Bfr with Mmar_3973. Inset in (A) shows the peaks corresponding to Mmar_3973 in the wash fraction. Panels (C) and (D) show equivalent fractions prepared using as isolated (24meric) Bfr. Masses of 7646 and 7515 Da are due to the ferredoxin Mmar_3973 (shown in red),^40^ whilst the predicted mass of the Bfr monomer is 18 596 Da (shown in black).

The His tag of ferredoxin encapsulated within Bfr would be unable to interact with the Ni^2+^ of the column and the protein would therefore elute in the wash step together with the ferritin. The LC-MS of the wash and elution fractions of the sample prepared as described above confirmed this to be the case (Fig. 5). The majority of the added ferredoxin remains free in solution and was detected in the elution fraction collected when stepping to an imidazole concentration of 500 mM. However, two peaks at masses of 7646 and 7515 Da were clearly visible in the mass spectrum of the fraction from the wash step (inset, Fig. 5A). These corresponded to the predicted mass of the Mmar_3973 peptide and that following loss of the N terminal methionine residue.^40^ Whilst the method is not rigorously quantitative, the relative peak heights are indicative of encapsulation of approximately 4 molecules of ferredoxin per Bfr cage.

That this is not the result of non-specific interactions between the exterior of the Bfr cage and Mmar_3973 that might render the His tag inaccessible to the Ni^2+^ column was confirmed by analysis of a sample prepared in parallel using 1 mg mL^−1^ Bfr in MES pH 6.5 that had not been dissociated into dimeric subunits (Fig. 5C). In this sample there is no evidence of the presence of Mmar_3973 in the fraction from the wash step, with ferredoxin only detected once the imidazole concentration had been increased to 500 mM (Fig. 5D).

**Fig. 6:**
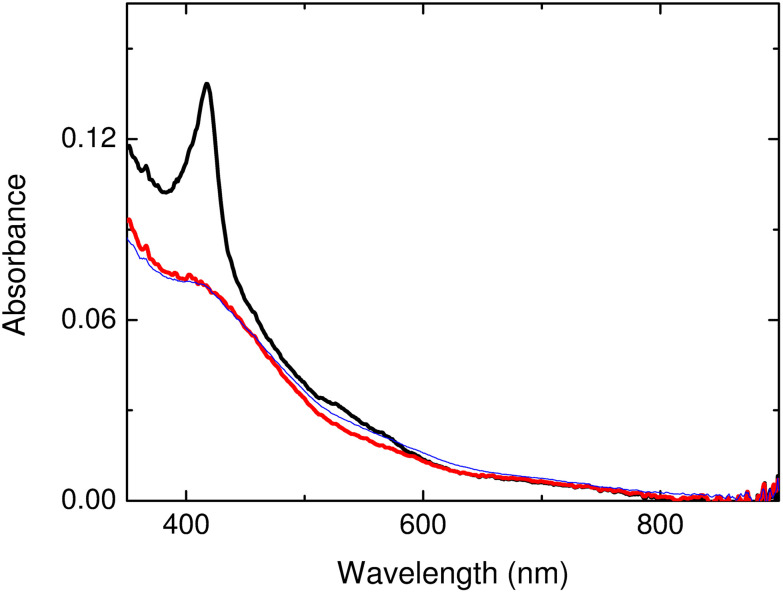
Absorbance of ferredoxin encapsulated within Bfr. Spectra of the encapsulated sample (heavy black trace), the same sample but with the contribution from the heme of Bfr subtracted (heavy red trace), and the ferredoxin starting material (blue trace). The latter overlays with the red trace, showing that the [3Fe-4S] cluster is intact. The sample contained ∼46 μM ferredoxin and ∼8 μM Bfr cages, indicating an average of ∼6 ferredoxin molecules per cage. All measurements were in a 1 mm pathlength cuvette.

UV-visible absorbance spectra of the wash fraction clearly indicated the presence of bands associated with the [3Fe-4S] cluster of the ferredoxin, demonstrating not only encapsulation, but also that the ferredoxin remained folded with its cluster intact and with no changes in redox state (Fig. 6). Subtraction of intensity due to Bfr enabled the quantification of Mmar_3973,^40^ indicating the presence of ∼6 ferredoxin molecules per Bfr 24mer.

Size exclusion chromatography was also used to separate Bfr from non-encapsulated ferredoxin. The absorbance spectrum and LC-MS of the Bfr-containing fraction (Fig. S4†) demonstrated co-elution of encapsulated ferredoxin, confirming the results of the Ni^2+^ affinity chromatographic experiments above. Finally, although re-cycling of non-dissociated Bfr was not employed during ferredoxin encapsulation trials, the yield of ferritin recovered from the process was still around 50%, considerably greater than typically achieved with acid dissociation.

## Discussion

The data presented demonstrate that the reversible dissociation of *E. coli* Bfr into subunit dimers can be performed *in vitro* under relatively mild conditions compared to those required for animal ferritins, and similar to those reported for the Ftn proteins from *A. fulgidus* and *T. maritima.*^15,17,32^ Dissociation of the protein cage is promoted by exchanging into low ionic strength buffer at pH 8.5–9.0, *i.e.* low metal and proton concentration, and can be reversed by returning to near neutral pH in the presence of a high concentration (1.5 M) of NaCl. The co-existence of dimeric and fully assembled wild type Bfr in solution has been known for some time and previous studies have identified key residues controlling protein association.^43,46,47^ Replacement of these resulted in subunit dimers with impaired ability to assemble into fully formed protein cages. Several of these residues, Arg30, Lys33, Arg61, Glu128 and Glu135, contain sidechains which undergo a change in protonation state at extremes of pH. However, the degree of dissociation of Bfr into subunit dimers as a function of pH is not described by the Henderson-Hasselbach equation (Fig. 2), and the p*K*_a_ values of the basic residues listed above (12.5 for Arg and 10.5 for Lys) are well above the pH at which significant dissociation of the protein cage occurs. It therefore seems unlikely that the association state of the protein is controlled simply by protonation/de-protonation events. Electrostatic interactions between subunits, mediated by metal ions, have been reported to affect the kinetics of protein association in other ferritins^27,48^ and the data presented here suggest that, in the case of *E. coli* Bfr, this effect also influences the extent of cage formation, possibly by mediating favourable electrostatic interactions within the channels penetrating the protein coat, or in the water filled pockets that are absent from animal ferritins. It seems likely that, rather than being determined simply by pH, Bfr association state is determined by a complex interplay of pH, metal ion concentration, temperature and hydrostatic pressure.^42,49^ In the case of the Ftns from *A. fulgidus* and *T. maritima*, an Asp residue (Asp65) that is not generally conserved (and which is not conserved in *E. coli* Bfr) was found to play a key role in modulating the proteins’ assembly state response to salt concentration.^50,51^ Therefore, whilst the conditions required to induce dissociation of Bfr are similar to those for the Ftns the physical basis for the change in association state appears to differ between the two classes of ferritin. Our data suggest that one consequence of this is a significantly reduced rate of inter-conversion between the oligomeric forms of Bfr. For example, the simple mixing of protein with heme (for which a high affinity binding site exists on the inner surface of the Bfr cage) does not lead to significant heme binding (*i.e.* successful encapsulation).

Ferritins are considered promising candidates for the encapsulation of drugs and other medically important compounds, such as fluorescent probes or contrast agents for imaging. The tendency of many malignant cells to overexpress receptors for ferritin uptake is beneficial for the targeting of these compounds,^5,45^ whilst encapsulation leads to lower general toxicity toward healthy tissue and increases the circulation time within the body. Whilst several demonstrations of the technical feasibility of these strategies have been reported, these have not yet resulted in the transition to clinical applications. One of the factors limiting the implementation of medical applications of encapsulating molecules within ferritin cages is the cost relative to existing technologies. The low yield of protein recovered from acid/base dissociation is a significant contribution to the cost of this process. Whilst it has been demonstrated that the yield of recovered protein can be increased by working under high pressure,^49^ this would also lead to significant additional costs for the production of large amounts of material. Thus understanding and exploiting the factors determining the conditions under which ferritin cages can be disassembled will be advantageous to their exploitation in such applications.

Here we show that *E. coli* Bfr can be disassembled into subunit dimers under relatively mild conditions and that the slow inter-conversion of the different oligomeric forms makes their separation using size exclusion chromatography practical. In this way, wild type protein can be obtained in the purely dimeric form and controllably reassembled by a combination of lowering the pH to near neutral and increasing salt concentration, offering the potential for molecules to be trapped within the interior of the Bfr cage. The modulation of Bfr assembly state under mild conditions and the potential to collect and recycle non-dissociated protein from the size exclusion column minimises losses caused by protein unfolding.

Encapsulation of proteins within ferritin cages has been reported for horse spleen ferritin (employing a low pH method) with an artificial streptavidin-based transfer hydrogenase,^16^ and for the Ftns from *A. fulgidus* and *T. maritima.*^15,17,32,52^ For the latter, there is an apparent requirement for the encapsulated protein to carry a significant positive charge, suggesting that complementarity with the negatively charged inner surface of the protein is a key requirement. Here we demonstrate capture of hemin from solution by dimeric Bfr which can then be returned to the fully assembled form whilst retaining heme bound to the inner surface of the cage. This strategy exploits the natural heme binding site to anchor the encapsulation target to the inner surface of the disassembled cage in analogous fashion to the charge complementarity reported in the Ftn examples.

This approach also offers a potential route for encapsulation of molecules within a ferritin cage that are sensitive to extremes of pH. To explore this, we encapsulated the [3Fe-4S] cluster-containing ferredoxin Mmar_3973 within the internal cavity of Bfr. The data indicate that, on average, each Bfr cage contained 4–6 copies of the ferredoxin, accounting for approximately 25–35% of the total void space within the Bfr nanocompartment. The encapsulation of a negatively charged ferredoxin (predicted pI of 3.7^40^) indicates that charge complementarity is less important for Bfr encapsulation. Further investigation is required but one possibility is that the high salt concentrations (similar to those used for precipitants in protein crystallography screens) lead to charge compensation on the negatively charged ferredoxin. The presence of the hydrophobic heme binding pocket, unique to Bfrs amongst ferritins, may then result in favourable interaction between ferredoxin and Bfr such that the cargo protein is associated with the inner surface of the cage upon reassembly. Nevertheless we demonstrate encapsulation of a protein with a chemically fragile [3Fe-4S] cofactor intact, expanding the scope for the use of ferritin as a bioreactor. In this respect, in contrast to the Ftns, encapsulation and retention of cargo proteins within Bfr is favoured by physiological conditions of neutral pH and moderate salt concentration where encapsulated enzymes would be expected to have optimum activity. This, combined with the presence of both acidic and hydrophobic regions on the inner surface of Bfr, may result in it being a more flexible platform for applications as a bioreactor nanocompartment. Whilst in principle proteins could become encapsulated within assembling ferritin cages *in vivo* there is no evidence to suggest that this is the case. To date the only substances identified within the interior of ferritins as isolated are hydrated ferric-oxo minerals together with varying levels of phosphate.

## Data availability

All data are available from the corresponding authors upon request.

## Author contributions

J. M. B.: conceptualization, investigation, formal analysis, writing – original draft. E. G. investigation, formal analysis, writing – review and editing. J. R. investigation, formal analysis. G. R. M.: conceptualization, supervision, writing – review and editing. N. L. B.: conceptualization, funding acquisition, supervision, writing – review and editing.

## Abbreviations

Bfr: Bacterioferritin
ESI-LC-MS: Electrospray ionisation – liquid chromatography – mass spectrometry
EDTA: Ethylene diamine tetraacetic acid
HEPES: 4-(2-Hydroxy ethyl)-1-piperazine ethane sulfonic acid
IMAC: Immobilised metal affinity chromatography
MES: 2-(*N*-morpholino) ethane sulfonic acid
PAGE: Polyacrylamide gel electrophoresis
SDS: Sodium dodecyl sulfonate
TEM: Transmission electron microscopy
Tris: Tris(hydroxymethyl)aminoethane.

## Conflicts of interest

The authors declare that there are no conflicts of interest associated with the manuscript.

## Supplementary Material

NR-014-D2NR01780F-s001
