# Supplementary material for: Protein encapsulation within the internal cavity of a bacterioferritin
Source: Nanoscale. 2022 Aug 15;14(34):12322–31. doi: 10.1039/d2nr01780f (PMC9439638; doi:10.1039/d2nr01780f)
Supplement: NR-014-D2NR01780F-s001 [file NR-014-D2NR01780F-s001.pdf]

Electronic Supplementary Material (ESI) for Nanoscale.
This journal is © The Royal Society of Chemistry 2022

Supporting Information

Protein encapsulation within the internal cavity of a bacterioferritin

Justin M. Bradley, Elizabeth Gray, Jake Richardson, Geoffrey R. Moore and Nick E. Le Brun

Supporting Figures

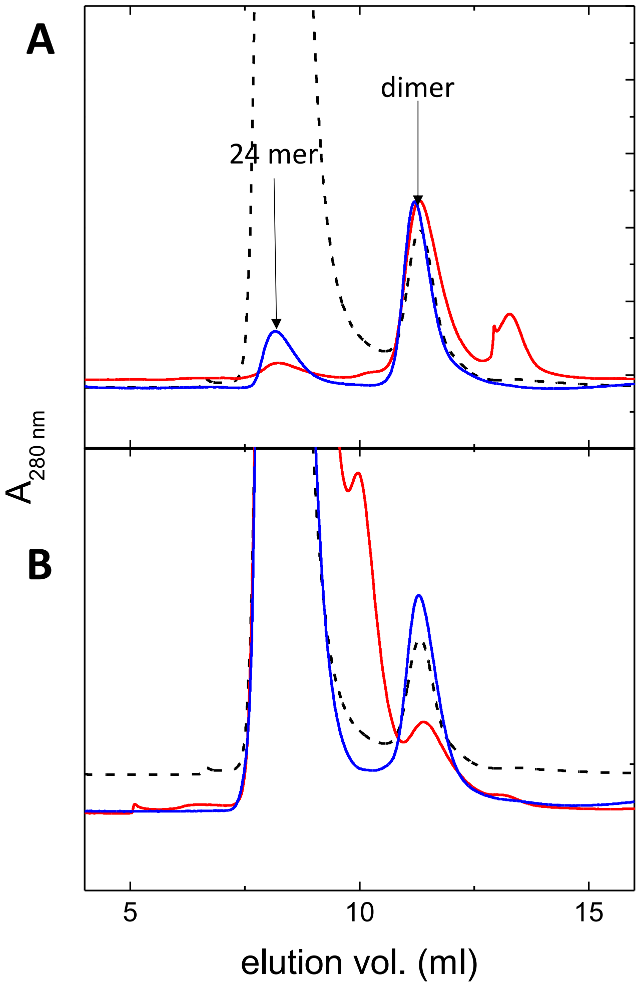

Figure S1. Equilibration of Bfr association states. Elution profiles from a Superdex 75 10/300 GL analytical gel filtration column equilibrated with 100 mM MES pH 6.5 of (A) samples prepared from protein eluting at the volume predicted for dimeric Bfr following incubation at 4 ˚C for 24 hours (red trace) or 7 days (blue trace). (B) as in (A) but using protein eluting at the volume predicted for 24meric Bfr. To allow comparison of samples with large differences in concentration traces in (A) are scaled such that the area under the peaks eluting at 11.5 mL are approximately equal showing the extent to which intensity at 8 mL is recovered. Similarly traces in (B) are scaled such that the peak heights at 8 mL are approximately equal allowing comparison of the relative peak areas at 11.5 mL. The sample eluting at the volume predicted for the 24mer likely contains oligomeric states intermediate between 24mer and dimer following 24 hours incubation at 4 ˚C. The dashed black traces are the elution profile of the sample from which dimeric and 24meric fractions were originally isolated to demonstrate the extent of re-equilibration at each incubation time. The data demonstrate that re-equilibration of 24mer and dimer components back towards mixtures of 24mer/dimer takes place, but that this is very slow, and may not be complete even after 7 days. The sample of dimeric Bfr (A) contains a small peak eluting at 13 mL following 24 hours of equilibration. This corresponds to a mass of 18 kDa and is therefore most likely monomeric Bfr.

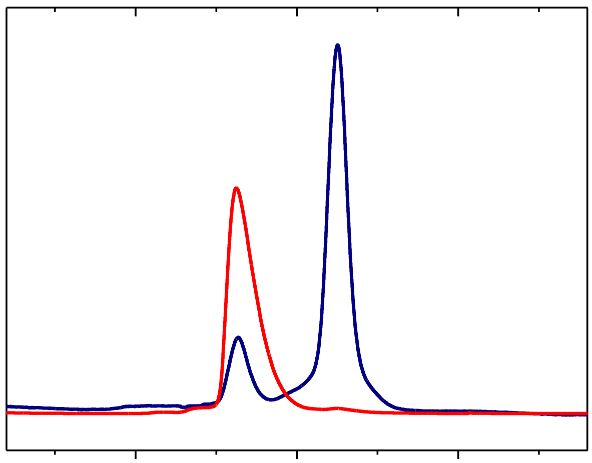

Figure S2. Re-assembly of dimeric Bfr into the fully assembled 24meric cage. Following incubation in 100 mM MES, 1.5 M NaCl pH 6.5 for 16 hours at 4 ˚C and exchange into NaCl free MES pH 6.5, Bfr elutes from the G75 analytical size exclusion column at the volume (8 mL) expected for the fully assembled cage (red trace). Blue trace shows the elution profile of Bfr incubated overnight in 20 mM Tris pH 9.0 from the Superdex 75 10/300 GL analytical gel filtration column equilibrated with the identical buffer.

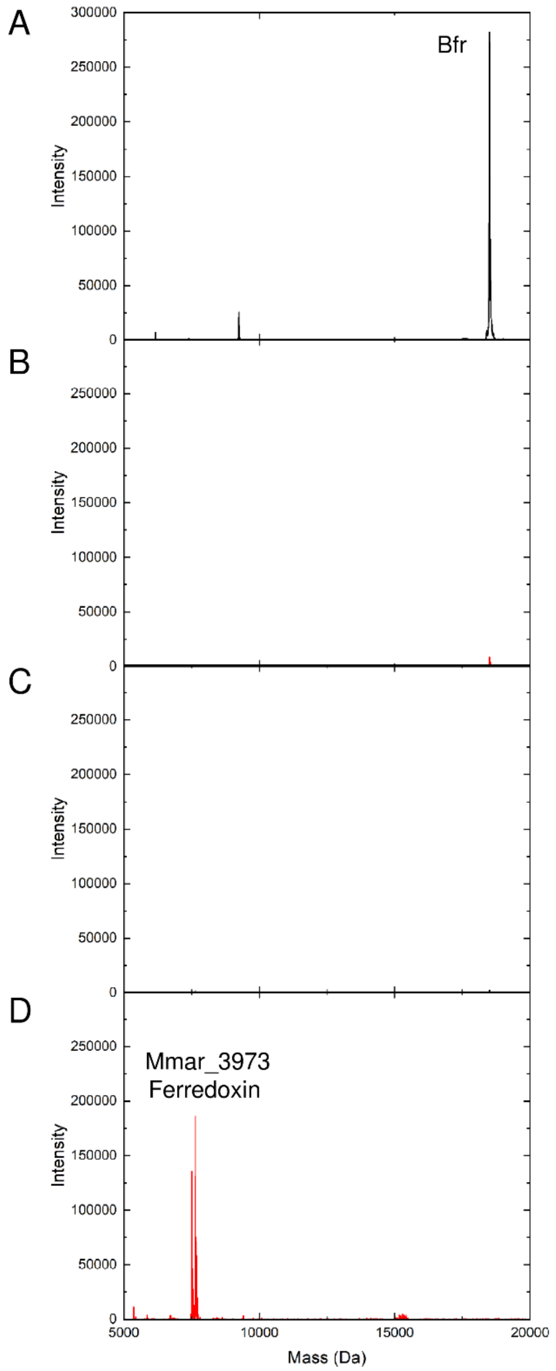

Figure S3. Demonstration of complete binding of Mmar_3973 and negligible binding of Bfr to a Ni^{2+} affinity column. The mass spectra of fractions collected during the wash (A) and elution (B) steps following the addition of Bfr in 20 mM Tris pH 9.0 mixed with an equal volume of 100 mM MES, 3 M NaCl pH 6.0 to the column. (C) and (D), as (A) and (B) but following the addition of Mmar_3973 in 100 mM MES, 3 M NaCl pH 6.0 mixed with an equal volume of 20 mM Tris pH 9.0 to the column. Bfr (18495 Da, shown in black) is detected only in the wash fraction, demonstrating negligible interaction with the column. Mmar_3973 (7646 and 7515 Da, shown in red) is detected only upon developing the column with elution buffer, demonstrating negligible flow through of the His tagged protein.

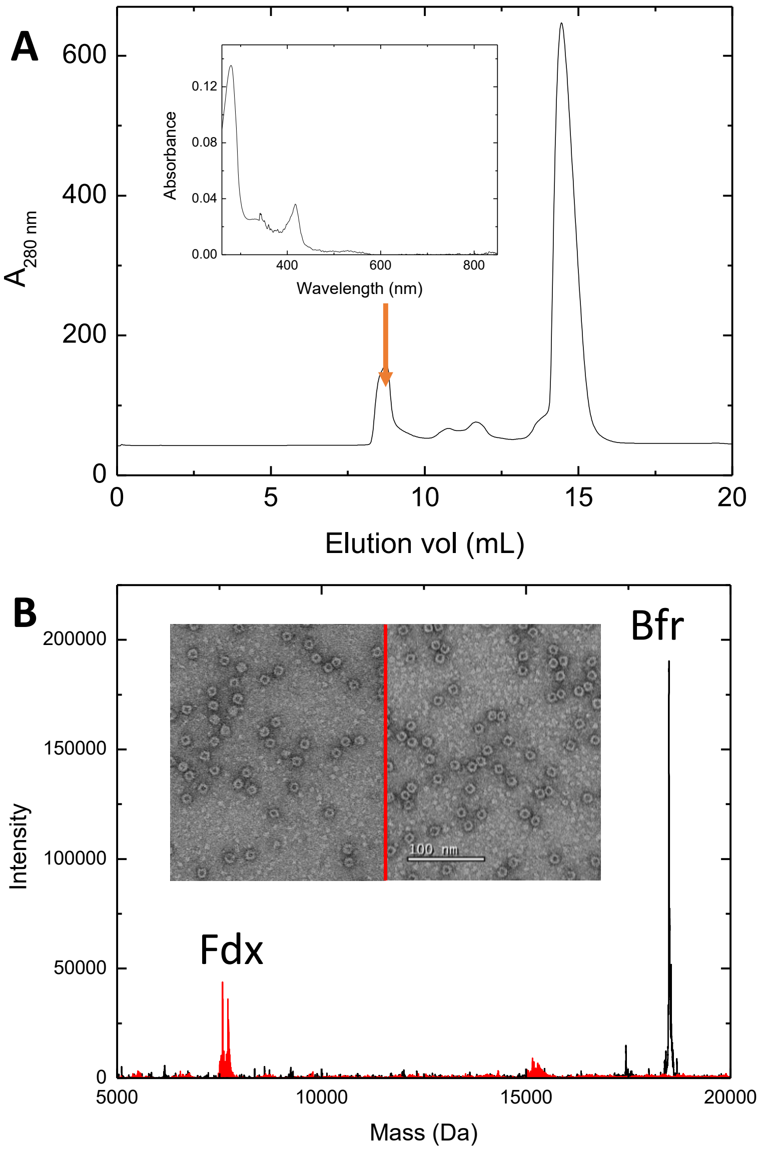

Figure S4. Size exclusion chromatography of Bfr encapsulated Fdx. Panel (A) shows the elution profile from a Superdex 75 10/300 GL analytical gel filtration column, equilibrated with 100 mM MES pH 6.5, 1.5 M NaCl, of a sample prepared by equal volume mixing of 1 mg mL^{-1} Bfr in 20 mM Tris pH 9.0 with 10 mg mL^{-1} Fdx3973 in 100 mM MES pH 6.0, 3 M NaCl. Inset shows the absorbance spectrum of the Bfr-containing fraction, indicated by the red arrow, that elutes at 8 mL. The large feature at 14.5 mL is non-encapsulated ferredoxin. (B) Mass spectrum of the 24meric Bfr eluting at 8 mL showing the presence of both Bfr (18495 Da shown in black) and Fdx3973 (predicted masses of 7646 and 7515 Da shown in red). We note that despite the column being developed using deoxygenated buffer some oxidative damage of the ferredoxin is evident (adducts bearing additional masses of 16 Da) which most likely occurred during fraction collection. Inset in panel B shows TEM images collected on Bfr as isolated (left) and following the encapsulation process (right) demonstrating reassembly of the 24mer protein cage.
